# Polymorphisms of *Interleukin-6* and *Interleukin-8* Are Not Associated with Parkinson’s Disease in Taiwan

**DOI:** 10.3390/brainsci11060768

**Published:** 2021-06-09

**Authors:** Tsai-Wei Liu, Yih-Ru Wu, Yi-Chun Chen, Hon-Chung Fung, Chiung-Mei Chen

**Affiliations:** 1 Department of Neurology, Chang-Gung Memorial Hospital, 5 Fuhsing St., Gueishan, Tauoyan 333, Taiwan; wusmallpu@gmail.com (T.-W.L.); yihruwu@cgmh.org.tw (Y.-R.W.); asd108@cgmh.org.tw (Y.-C.C.); 2 Department of Neurology, College of Medicine, Chang-Gung University, 259 Wen-Hwa 1st Rd., Kwei-Shan Dist, Tauoyan 333, Taiwan; 3 Fu Jen Faculty of Theology of St. Robert Bellarmine, Fu Jen University Clinic, Zhongzheng Rd., Xinzhuang Dist., New Taipei City 242, Taiwan; pf200272@hotmail.com

**Keywords:** inflammation, *IL-6*, *IL-8*, Parkinson’s disease, genetic polymorphism, disease association

## Abstract

Background: Studies have suggested that cytokines are crucial mediators in the pathogenesis of Parkinson’s disease (PD). The multifunctional cytokine interleukin (IL)-6 and its single nucleotide polymorphisms (SNPs) were found to have an impact on the development of PD. However, different studies in associations of *IL-6* genetic variants with PD showed inconsistent results and it has never been explored in a Taiwanese population. Both IL-1α and IL-8 contribute to the same inflammation pathway. *IL-1α* genetic polymorphism has an effect on late-onset PD in Taiwan, whereas the associations of *IL-8* genetic variants with PD in Taiwan remain to be investigated. Methods: This study examined the frequencies of polymorphisms within the critical promoter areas of the proinflammatory cytokine genes: *IL-6* G-174C (rs1800795) and *IL-8* A-251T (rs4073) in Taiwanese PD patients compared with age-and gender-matched healthy subjects. Comparisons were also made in genotype and allele frequencies of *IL-6* G-174C (rs1800795) and *IL-8* A-251T (rs4073) among different populations in previous studies. Results: In total, 1120 subjects, including 509 PD patients (female/male: 259/250) and 511 control subjects (female/male: 252/259), were recruited. We found no statistically significant differences in *IL-6* G-174C (rs1800795) or *IL-8* A-251T (rs4073) genotypic and allelic distribution between PD and controls, even after being stratified by age at onset and gender. Conclusions: The results did not demonstrate any association of *IL-6* G-174C (rs1800795) or *IL-8* A-251T (rs4073) with PD in a Taiwanese population. Despite the negative results, this is the first study in associations of *IL-6* G-174C (rs1800795) and *IL-8* A-251T (rs4073) with PD in Taiwan. The relevance of genetic variants of *IL-6* G-174C (rs1800795) or *IL-8* A-251T (rs4073) on PD susceptibility warrants further investigation.

## 1. Introduction

Parkinson’s disease (PD) is one of the common neurodegenerative diseases. While PD is featured by the progressive accumulated Lewy bodies with diminished dopaminergic neurons in the substantia nigra par compacta (SNpc), the pathogenesis is complex. McGeer and colleagues showed the premier evidence of activated microglial cells in the SNpc of post-mortem PD brain [1]. The presence of neuroinflammatory processes in the post-mortem brains of PD patients has been further proved at a molecular level [1]. Mogi and colleagues addressed an increment in levels of epidermal growth factor (EGF), transforming growth factor α (TGFα), interleukins (IL)-1β, and IL-6 in the striatum and cerebrospinal fluid (CSF) of PD patients [2,3]. There have been growing studies focusing on the correlation between peripheral inflammation and neurodegenerative diseases, including PD, since the last decade [4,5,6,7,8]. A meta-analysis study demonstrated higher concentrations of IL-6, tumor necrosis factors (TNFs), IL-1β, IL-2, IL-10, C-reactive protein, and Regulated upon Activation, Normal T cell Expressed and presumably Secreted (RANTES) in peripheral blood of PD patients [9]. On the contrary, levels of interferon (IFN)-γ and IL-8 in PD patients were not distinctive from those in healthy controls [9]. Since the inflammatory responses caused by cytokines may be crucial in the pathogenesis of PD, numerous studies have addressed the genetic influence of inflammatory cytokines on the risk of PD. A meta-analysis [10] of genetic association studies suggested that gene polymorphisms of tumor necrosis factor-α (*TNF*α)-1031 (rs1799964), *IL-6* G-174C (rs1800795), and *IL-1RA* Variable number tandem repeat (VNTR) (rs2234663) could have an influence on PD risk. In contrast, no significant associations of *IL-1β* C-511T (rs16944), *IL-1α* C-889T (rs1800587), *TNFα* G-308A (rs1800629), and *IL-10* G-1082A (rs1800896) with PD were found [10]. Among them, the *IL-10* G-1082A (rs1800896) and *IL-6* G-174C (rs1800795) were examined only in the Caucasian population [10]. Nevertheless, *IL-1α *C-889T (rs1800587) has an influence on late-onset PD (LOPD) in Taiwan [11], whereas *IL-10* (-819 T/C) is a risk factor of EOPD and female PD patients and *IL-18* 607C/A (rs1946518) was associated with sporadic LOPD in Han population [12,13]. The ethnic differences of the studied populations may possibly explain the inconsistent results.

IL-6, a cytokine with multiple functions, is increased in striatal tissue, cerebrospinal fluid, and peripheral blood of PD patients [3,14,15,16,17]. A few reports have shown that a G/C single nucleotide polymorphism (SNP) at position-174 *(*rs1800795) affects the IL-6 expression level, but different studies have debated on which genotype is linked to a higher expression level [18,19,20,21]. Nevertheless, the G allele and GG genotype have been previously shown to increase the risk of PD, especially in early-onset patients from Sweden [22].

IL-8, a kind of chemokines, is known as CXCL8. *IL-8* gene is positioned in chromosome 4q13-21, where the familiar PD-causing genes, α*-synuclein* (4q-21), *UCH-L1* genes (4q14-15), and the *PARK4* loci (4q15), are also located. As located at a similar locus with those causative genes of familial PD, *IL-8* was suggested to be possibly associated with familial PD [23]. Despite that the association of a functional promoter region polymorphism (−251) of the *IL-8* gene with PD has been lately noted in the Irish population [24], the Spanish study showed a negative result [25]. Furthermore, the Turkey [26] study found the uncertain role of the *IL-8* A-251T (rs4073) gene in PD because TT and TA allele frequencies were similar between PD patients and control subjects, which were different from those in the Irish population. Accumulated evidence has shown that IL-1α and IL-8 share the same pathway in the inflammatory response, and a combination of these two genes has contributed to an increased risk of Alzheimer’s disease (AD) [27]. Previously, our study has shown that subjects carrying *IL-1α *C-889T (rs1800587) T allele may exert a protective effect against late-onset PD (disease onset > 70 years) [11]. The *IL-6* G-174C (rs1800795) and *IL-8* A-251T (rs4073) may be associated with the risk of PD, but only a limit of studies with small numbers of samples have been conducted to determine the genetic influence worldwide [7,10]. Although several studies have demonstrated genetic associations of cytokines with PD, associations were not found for specific cytokines and across different ethnic groups [10].

Since associations of PD with *IL-6* and *IL-8* SNPs have never been examined in Taiwanese, our objective in this paper is to assess the SNPs of cytokines, *IL-6* G-174C (rs1800795) and *IL-8* A-251T (rs4073), in PD, particularly in Taiwan population.

## 2. Materials and Methods

### 2.1. Ethics Statement

This study was conducted according to the protocol approved by the Institutional Review Board of Chang Gung Memorial Hospital (ethical license No.: 102-5614A3 and 201701921A3). All laboratory works were performed after receiving informed consent from all the patients and controls.

### 2.2. Subjects

In total, 1017 Taiwanese subjects, including 508 PD patients and 509 unrelated age-matched controls, were genotyped for *IL-6* G-174C (rs1800795). And 1016 Taiwanese subjects, including 508 PD patients and 508 unrelated age-matched controls, were genotyped for *IL-8* A-251T (rs4073). All patients were examined by two movement disorder specialists (Y.-R.W. and C.-M.C.) and diagnosed with PD based on the United Kingdom PD Society Brain Bank clinical diagnostic criteria [28]. The disease stage was assessed in line with Hoehn and Yahr stages. Unrelated healthy individuals with matched age, gender, ethnic origin, and area of residence were recruited from the outpatient clinic of Chang Gung Memorial Hospital. Patients with age at onset of below 50 years were classified as early-onset PD (EOPD), while subjects with an age at onset above 50 years were classified as late-onset PD (LOPD).

### 2.3. Genetic Analysis

SNP *IL-6,* rs1800795, and *IL-8*, rs4073, were selected from the GWAS meta-analysis in the PD Gene database (http://www.pdgene.org/gwas (accessed on 9 June 2021)). The SNP genotyping was performed according to the methods described in our previous works [29]. The sequences of specific polymerase chain reaction (PCR) (*IL-6* G-174C, rs1800795, forward: GAACACAGAAGAACTCAGATGACTGG reverse: AGGAGTTCATAGCTGGGCTCTGGAG; *IL 8* A-251T, rs4073, forward: CTTATCTTCACCATCATGATAGCATCTG, reverse: GGCTGCCAAGAGAGCCACGGCCAGC) were designed with Assay Designer (version 4.0) (Agena, San Diego, CA, USA).

### 2.4. Statistical Analysis

The frequencies of genotypes and alleles between PD patients and healthy controls were compared using the Pearson χ^2^ test. Odds ratios (OR) with 95% confidence interval (CI) for genotyping were calculated. Statistically, significance was considered when a *p*-value was less than 0.05. Hardy–Weinberg equilibrium for genotype frequencies of the patients and controls was checked using an exact test. The power of the study was calculated by using software G*power 3.1. This study had a power greater than 0.8 when the odds ratio (OR) of the per-allele genetic effect was greater than 1.4 or lesser than 0.70 for both rs1800795 and rs4073 when the observed allele frequency was at the significance level of 0.05.

## 3. Results

The genotype distributions in PD patients and controls did not depart significantly from Hardy-Weinberg equilibrium for all the polymorphisms examined (data not shown). For rs1800795 genotyping, the mean age and sex (female/male) ratios were respectively 63.6 ± 10.7 years and 259/249 in patients with PD and 63.4 ± 11.9 years and 251/258 in controls. For rs4073 genotyping, the mean age and sex (female/male) ratio were respectively 63.5 ± 10.7 years and 259/249 in patients with PD and 63.4 ± 11.9 years and 251/257 in controls (Table 1). The genotype and allele frequency distributions of SNPs tested in PD patients and controls are shown in Table 2.

Neither the genotypic nor the allelic frequencies of rs1800795 or rs4073 polymorphism in PD patients were statistically different from those in the controls. Further stratification of the participants based on the onset age and gender did not show significant differences in allelic and genotypic frequencies of rs1800795 or rs4073 between PD patients and controls. Table 3 presents the characteristics of the previous *IL-6* and *IL-8* polymorphism studies in different countries.

## 4. Discussion

Our study found no association of *IL-6* G-174C (rs1800795) and *IL-8* A-251T (rs4073) with PD susceptibility in the Taiwan population. When PD patients were stratified according to gender or age, no obvious associations were observed as well. The results reported here do not support the findings of the previous studies on the impact of genetic variants of *IL-6* or *IL-8* on PD risk (Table 3a,b). A significantly higher frequency of the GG genotype of the *IL-6* G-174C was found in the PD patients, particularly at the early age of onset (younger than 50 years) of PD in Sweden [22]. In the Sweden population, the frequency of GG genotype also appears to be increased in late-onset PD patients compared to the controls, although this difference did not reach a statistical significance [22]. However, our cohort did not show a similar GG genotype distribution to that of the Sweden population in either late or early onset of PD (EOPD vs. controls: 100% vs. 100%; LOPD vs. controls: 99.6% vs. 99.8%). The study done by Luciano and colleagues supports the assumption that the *IL6* G−174C G allele serves as a gain-of-function variant causing a proinflammatory state associated with PD patients in Ashkenazi Jewish [30]. They also report different gender contributing to the risk SNP in a Non-Jewish Caucasian population [30]. However, we did not find a differential gender effect of *IL-6* SNP in our cohort. The increased frequency of GG genotype in PD compared with the controls is not replicated in Irish [24], Spanish [25], and Slovenia [31] samples. These three studies did not compare between patients with an early age of onset and age-matched controls. Only the Irish study made a comparison by gender stratification, though no statistical significance was found. Taken together, the association of *IL-6* G-174C with PD is controversial. One of the reasons for the inconsistent results among ours and previous studies was possibly due to various ethnics of the studied populations as genotypic distributions of cytokine genes have been proved to diverge among different geographical populations [32]. For instance, Caucasians in Northern Ireland have a lower *IL-6*-174 G allele frequency compared with other populations (54% compared with 100% of Chinese in Singapore and 99% of Chinese in Taiwan) [32]. The distinct genetic backgrounds in different populations suggest the differential effects of inflammation-related genetic variants on PD risk.

*IL-6* polymorphisms also have a role in AD-related immune reactions and are involved in neuronal damage during the disease progression [33]. Studies have shown higher levels of proinflammatory cytokines in peripheral blood of patients with AD or parkinsonism compared to controls [34]. However, the results of association studies of *IL-6* G-174C with PD in most populations were negative. The possible explanations may be the presence of other SNPs in tight linkage disequilibrium with the C/G SNP, which may distinctively influence the IL-6 level that varies with different populations.

Regarding the *IL-8* A-251T (rs4073), since our study showed negative results, we made comparisons in genotype and allele frequencies between different populations in previous studies. Ross and colleagues [24] showed a significantly lower TT frequency and a higher TA frequency in PD patients compared to the controls, suggesting that TT genotype may confer protection against the development of PD or affect disease pathogenesis. Because of the small sample size (*n* = 90 in PD; *n* = 93 in NC), the statistical power of the study on the Irish population conducted by Ross et al. is small and the significance is lost after correction for multiple comparisons [24]. However, the results may implicate that low IL-8 producer TT genotype is protective against the onset of PD. On the contrary, the *IL-8* TA genotype may render the individuals more vulnerable to PD development. Although there is a tendency toward increased TA genotype and decreased TT genotype frequency in our PD patients, it did not reach a statistical significance. The gene set-association analysis conducted by Infante et al. did not reveal the interactive effect of a cluster of five genes TNF-*α*, IL-6, IL-8, IL-1*α*, and IL-10 on the PD risk. Therefore, they suggested that the negative result may be due to the complex pathogenesis attributed to cytokines and genetic interactions between polymorphisms within the cytokine genes and genes nearby [25]. Furthermore, frequencies of TT and TA in PD patients were not different from those in the controls in the Turkey population [26]. Despite the possible associations of *IL-8* variants with PD suggested by the above studies, our study did not show the association.

IL-8, a key mediator associated with inflammation, is mainly functioning in neutrophil recruitment and degranulation. The secretion of IL-8 is increased by environmental stimulation, such as oxidative stress. However, the role played by IL-8 in dopaminergic cell death is still unclear [7]. IL-8 level is rapidly enhanced by proinflammatory cytokines such as IL-1α and IL-1α can upregulate IL-8 by more than 100-fold [35,36]. Evidence from literature has shown that IL-1α and IL-8 may be involved in the same pathway in the cerebral inflammatory reaction [27]. *IL-1A* (-889) T allele has been considered to be a risk factor for AD [37]. A study found that the cases carrying both the *IL-1A* (-889) allele T and the *IL-8* (-251) T/T genotype had twice the chance of developing AD than those without [27]. The above evidence may explain that both *IL-1α* and *IL-8* contribute to susceptibility to AD [27]. In contrast, previously we have demonstrated that in Taiwanese patients, bearing of *IL-1α*-889 T allele may have a lower risk of developing late onset PD (older than 70 years) [11]. Hull et al. also mentioned that the *IL-8*-251 T allele could influence *IL-*8 production directly by affecting the binding of transcription factors to the promoter region of the gene, or it may be in linkage disequilibrium with a functional variant away from *IL-8* or a nearby gene [38]. Therefore, the single-gene association studies in PD risk might often yield negative results [25,26]. Despite adequate statistical power, we observed no association of *IL-6* G-174C or *IL-8* A-251T in our PD patients in Taiwan. One of the reasons may include the genomic varieties in different ethnicities and various environmental factors in different geographical regions. In addition, we did not exclude any association of other SNPs near or within the gene. Besides, interactions of gene-gene and gene-environmental factors were not evaluated. A more extensive series of case-control studies in various ethnic populations shall be necessary to support these results.

## 5. Conclusions

Although our study results did not prove the association of *IL-6* G-174C or *IL-8* A-251T with PD in the Taiwan population, it is important to point out that the effects of genetic variants of cytokines on inflammatory responses in PD are complex. Since our results are different from previous studies reporting positive results, how genetic effects of cytokines, *IL-6* and *IL-8*, contribute to the risk of PD needs future studies to clarify further.

## Figures and Tables

**Table 1:** Demographic and clinical characteristics of Parkinson’s disease (PD) patients and controls.

	PD	Controls	Total	*p* Value
Number	509	511	1020	
Age (years)	63.6 ± 10.7	63.4 ± 11.9	63.5 ± 11.3	0.76
	(Age at onset)			
Gender (Female/male)	259/250	252/259	511/509	0.87

**Table 2:** Frequency of genotype and allele of *IL6* rs1800795 and *IL-8* rs4073 polymorphism among Parkinson’s Disease (PD) patients and controls in Taiwanese.

(**a**) Frequency of genotype and allele of *IL6* rs1800795 polymorphism among Parkinson’s Disease (PD) patients and controls in Taiwanese.						
**rs1800795, *IL-6* G-174C**	**Genotype/Allele**	**PD (%)**	**Control (%)**	**χ2**	**Odd Ratio**	***p*** **Value**
All (*n* = 1017)		508	509			
Genotype	GG	506 (99.6)	508 (99.4)	N/A	1	
	GC	2 (0.4)	1 (0.2)		2.008 (0.182~22.215)	0.569
	CC	0	0		N/A	N/A
Dominant model	GG	506 (99.6)	508 (99.4)		1	
	GC + CC	2 (0.4)	1 (0.2)		2.008 (0.182~22.215)	0.569
Recessive model	GC + GG	508 (100)	509 (100)		1	
	CC	0	0		N/A	N/A
Allele	G (major)	1014 (99.8)	1017 (99.9)		1	
	C (minor)	2 (0.2)	1 (0.1)		2.006 (0.182~22.257)	0.570
Male (*n* = 507)		249	258			
Genotype	GG	247 (99.2)	258 (100)	N/A	1	
	GC	2 (0.8)	0		N/A	N/A
	CC	0	0		N/A	N/A
Dominant model	GG	247 (99.2)	258 (100)		1	
	GC + CC	2 (0.6)	0		N/A	N/A
Recessive model	GC + GG	249 (100)	258 (100)		1	
	CC	0	0		N/A	N/A
Allele	G (major)	496 (99.6)	516 (100)		1	
	C (minor)	2 (0.4)	0		N/A	N/A
Female (*n* = 510)		259	251			
Genotype	GG	259 (100)	250 (99.6)		1	
	GC	0	1(0.4)		N/A	N/A
	CC	0	0			
Dominant model	GG	259 (100)	250 (99.6)		1	
	GC + CC	0	1(0.4)		N/A	N/A
Recessive model	GC + GG	259 (100)	251 (100)		1	
	CC	0	0		N/A	N/A
Allele	G (major)	518 (100)	500 (99.8)		1	
	C (minor)	0	1 (0.2)		N/A	N/A
EOPD (*n* = 107)		44	63			
Genotype	GG	44 (100)	63 (100)	N/A	1	
	GC	0	0		N/A	N/A
	CC	0	0		N/A	N/A
Dominant model	GG	44 (100)	63 (100)		1	
	GC + CC	0	0		N/A	N/A
Recessive model	GC + GG	44 (100)	63 (100)		1	
	CC	0	0		N/A	N/A
Allele	G (major)	88 (100)	126 (100)		1	
	C (minor)	0	0		N/A	N/A
LOPD (*n* = 910)		464	446			
Genotype	GG	462 (99.6)	445 (99.8)	N/A	1	
	GC	2(0.4)	1(0.2)		N/A	N/A
	CC	0	0		N/A	N/A
Dominant model	GG	462 (99.6)	445 (99.8)		1	
	GC + CC	2 (0.4)	1 (0.2)		0.519 (0.047~5.745)	0.593
Recessive model	GC + GG	464 (100)	446 (100)		1	
	CC	0	0		N/A	N/A
Allele	G (major)	926 (99.8)	891 (99.9)		1	
	C(minor)	2 (0.2)	1 (0.1)		0.520 (0.047~5.741)	0.593
(**b**) Frequency of genotype and allele of IL-8 rs4073 polymorphism among Parkinson’s disease (PD) patients and controls in Taiwanese.						
**rs4073,** ***IL*** ** - ** ***8*** **A-251T**	**Genotype/Allele**	**PD (%)**	**Control (%)**	**χ2**	**Odd Ratio**	***p*** **Value**
All (*n* = 1016)		508	508			
Genotype	TT	200 (39.3)	213 (41.9)	0.691	1	
	TA	227 (44.7)	217 (42.7)		1.114 (0.852~1.457)	0.430
	AA	81 (15.9)	78 (15.4)		1.106 (0.767~1.595)	0.59
Dominant model	TT	200 (39.4)	213 (41.9)		1	
	TA + AA	308 (60.6)	295 (58.1)		1.111 (0.866~1.428)	0.406
Recessive model	TA + TT	427 (84.1)	430 (84.6)		1	
	AA	81 (15.9)	78 (15.4)		1.046 (0.745~1.467)	0.796
Allele	T (major)	627 (61.7)	643 (63.2)		1	
	A(minor)	389 (38.3)	374 (36.7)		1.067 (0.891~1.277)	0.481
Male (*n* = 506)		249	257			
Genotype	TT	100 (40.2)	107 (41.6)	1.5	1	
	TA	117 (46.9)	109 (42.4)		1.148 (0.788~1.675)	0.472
	AA	32 (12.9)	41 (16)		0.835 (0.488~1.428)	0.511
Dominant model	TT	100 (40.2)	107 (41.6)		1	
	TA + AA	149 (59.8)	150 (58.4)		1.063 (0.746~1.515)	0.736
Recessive model	TA + TT	217 (87.1)	216 (84)		1	
	AA	32 (12.9)	41 (16)		0.777 (0.472~1.280)	0.322
Allele	T (major)	317 (63.7)	323 (62.8)		1	
	A(minor)	181 (36.3)	191 (37.2)		0.966 (0.748~1.247)	0.788
Female (*n* = 510)		259	251			
Genotype	TT	100 (38.6)	106 (42.2)	1.74	1	
	TA	110 (42.5)	108 (43.0)		1.079 (0.738~1.580)	0.694
	AA	49 (19)	37 (14.7)		1.404 (0.846~2.330)	0.189
Dominant model	TT	100 (38.6)	106 (42.2)		1	
	TA + AA	159 (61.4)	145 (57.8)		1.162 (0.816~1.656)	0.405
Recessive model	TA + TT	210 (81.1)	214 (85.3)		1	
	AA	49 (18.9)	37 (14.7)		1.350 (0.846~2.154)	0.209
Allele	T (major)	310 (59.8)	320 (63.6)		1	
	A(minor)	208 (40.2)	183 (36.4)		1.173 (0.911~1.511)	0.215
EOPD (*n* = 106)		45	61			
Genotype	TT	18 (40)	23 (37.7)	0.744	1	
	TA	21 (46.7)	26 (42.6)		0.968 (0.417~1.252)	0.942
	AA	6 (13.3)	12 (19.7)		0.639 (0.223~2.314)	0.579
Dominant model	TT	18 (40)	23 (37.7)		1	
	TA + AA	27 (60)	38 (62.2)		0.907 (0.412~1.020)	0.811
Recessive model	TA + TT	39 (86.7)	49 (80.3)		1	
	AA	6 (13.3)	12 (19.6)		0.628 (0.216~1.825)	0.810
Allele	T (major)	57 (64.8)	72 (9)		1	
	A(minor)	31 (35.2)	50 (41)		0.783 (0.4444~1.380)	0.398
LOPD (*n* = 910)		463	447			
Genotype	TT	182 (39.3)	190 (42.5)	1.03	1	
	TA	206 (44.5)	191 (42.7)		1.125 (0.848~1.494)	0.411
	AA	75 (16.2)	66 (14.8)		1.186 (0.805~1.749)	0.388
Dominant model	TT	182 (39.3)	190 (42.5)		1	
	TA + AA	281 (60.7)	257 (57.5)		1.141 (0.876~1.487)	0.327
Recessive model	TA + TT	388 (83.8)	381 (85.2)		1	
	AA	75 (16.2)	66 (14.8)		1.116 (0.779~1.599)	0.550
Allele	T (major)	570 (61.6)	571 (63.9)		1	
	A(minor)	356 (38.4)	323 (36.1)		1.104 (0.913~1.335)	0.307

**Table 3:** Characteristics of the association studies of *IL-6* rs1800795 and *IL-8* rs4073 in PD.

(**a**) Characteristics of the association studies of IL-6 rs1800795 in PD.											
**First** **Author**	**Year**	**Country**	**Ethnicity**	**Genotype Method**	**Sample Size**	**Genotype Distribution (Case/Control)**				**MAF (Case/Control)**	**(HWE)**
					**Case/Control**	**GG**	**GC**	**CC**	**Minor Allele**		
Ross	2004	Ireland	Caucasian	PCR	90/93	26/32	44/50	11/20	C	0.477/0.387	Yes
Hakansson	2005	Sweden	Caucasian	PCR	265/308	78/68	129/162	51/78	C *	0.447/0.516	Yes
Infante	2008	Spain	Caucasian	Taq-Man SNP assay/ABI PRISM 7000 sequence detection system	196/170	88/62	81/81	27/27	C	0.344/0.397	Yes
Luciano	2012	USA	Caucasian	PCR	381/521	205/208	144/245	31/69	C *	0.271/0.366	Yes
Redensek	2019	Slovenia	Caucasian	TaqMan genotyping assays	224/146	65/45	120/67	39/34	C	0.441/0.462	Yes
Liu	X	Taiwan	Asian	PCR/Agena MassARRAY	508/511	506/508	2/1	0/0	C	0.003/0.001	Yes
(**b**) Characteristics of the association studies in IL-8 rs4073 in PD.											
**First** **Author**	**Year**	**Country**	**Ethnicity**	**Genotype Method**	**Sample Size**	**Genotype Distribution (Case/Control)**				**MAF (Case/Control)**	**(HWE)**
					**Case/Control**	**TT**	**TA**	**AA**	**Minor Allele**		
Ross	2004	Ireland	Caucasian	PCR	90/93	18/34	54/39	18/20	A *	0.5/0.424	Yes
Infante	2008	Spain	Caucasian	Taq-Man SNP assay/ABI PRISM 7000 sequence detection system	197/173	46/48	107/90	44/35	A	0.494/0.462	Yes
Calapoglu	2016	Turkey	Caucasian	PCR-RFLP	30/60	10/22	9/30	11/8	A	0.516/0.383	Yes
Liu	X	Taiwan	Asian	PCR/Agena MassARRAY	508/508	200/213	227/217	81/78	A	0.382/0.367	Yes

MAF: Minor Allele Frequency; HWE: Hardy-Weinberg equilibrium; PCR: polymerase chain reaction; PCR-RFLP: polymerase chain reaction/restriction fragment length polymorphism; All studies match Hardy-Weinberg equilibrium; *: Result shows a significant association with PD risk.

## Data Availability

The data presented in this study are available on sequent to corresponding author.
